# Pharmacogenomic Impact of *CYP2C19* Variation on Clopidogrel Therapy in Precision Cardiovascular Medicine

**DOI:** 10.3390/jpm8010008

**Published:** 2018-01-30

**Authors:** Sherry-Ann Brown, Naveen Pereira

**Affiliations:** 1 Department of Cardiovascular Diseases, Mayo Clinic, 200 First Street SW, Rochester, MN 55905, USA; 2 Department of Molecular Pharmacology and Experimental Therapeutics, Mayo Clinic, 200 First Street SW, Rochester, MN 55905, USA; pereira.naveen@mayo.edu

**Keywords:** pharmacogenomics, CYP450, genetics variants, antiplatelet therapy, clopidogrel, *CYP2C19*, precision cardiovascular medicine

## Abstract

Variability in response to antiplatelet therapy can be explained in part by pharmacogenomics, particularly of the CYP450 enzyme encoded by *CYP2C19*. Loss-of-function and gain-of-function variants help explain these interindividual differences. Individuals may carry multiple variants, with linkage disequilibrium noted among some alleles. In the current pharmacogenomics era, genomic variation in *CYP2C19* has led to the definition of pharmacokinetic phenotypes for response to antiplatelet therapy, in particular, clopidogrel. Individuals may be classified as poor, intermediate, extensive, or ultrarapid metabolizers, based on whether they carry wild type or polymorphic *CYP2C19* alleles. Variant alleles differentially impact platelet reactivity, concentration of plasma clopidogrel metabolites, and clinical outcomes. Interestingly, response to clopidogrel appears to be modulated by additional factors, such as sociodemographic characteristics, risk factors for ischemic heart disease, and drug-drug interactions. Furthermore, systems medicine studies suggest that a broader approach may be required to adequately assess, predict, preempt, and manage variation in antiplatelet response. Transcriptomics, epigenomics, exposomics, miRNAomics, proteomics, metabolomics, microbiomics, and mathematical, computational, and molecular modeling should be integrated with pharmacogenomics for enhanced prediction and individualized care. In this review of pharmacogenomic variation of CYP450, a systems medicine approach is described for tailoring antiplatelet therapy in clinical practice of precision cardiovascular medicine.

## 1. Introduction

**Figure 1:**
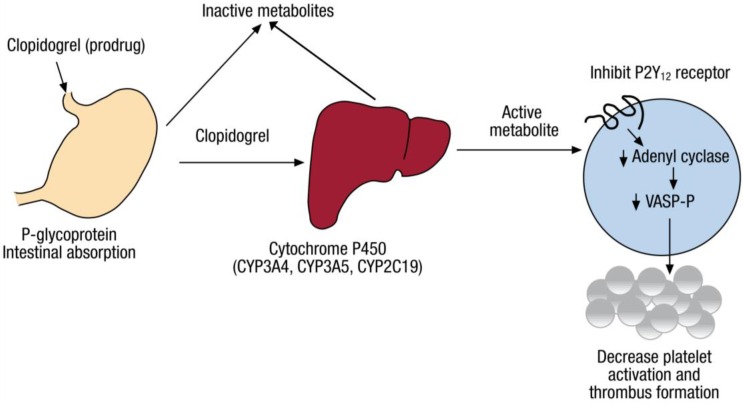
Metabolism of clopidogrel. The pharmacodynamics and pharmacokinetics of clopidogrel (a prodrug) involves intestinal absorption via P-glycoprotein. This is followed by processing of clopidogrel in the liver primarily by cytochrome P450 enzymes, with release of the active metabolite that inhibits the adenosine diphosphate P2Y12 receptor. This leads to decrease in activation of platelets and formation of thrombus. Used with permission of the Sociedad Española de Cardiología; copyright 2011 [22].

Clopidogrel is a commonly used antiplatelet drug; medical society consensus guidelines broadly recommend its use [1,2,3] for prevention of stent thrombosis in individuals treated with percutaneous intervention (PCI) [4,5], and in some patients medically managed after non ST elevation myocardial infarction. In PCI, a metal stent is inserted into a coronary artery to improve blood flow in patients presenting with ST elevation myocardial infarction, acute coronary syndrome (ACS), or refractory stable angina [6,7,8]. Clopidogrel is an oral second-generation thienopyridine with 50% bioavailability; its concentration peaks at 1 to 2 h following administration of an initial or “loading” dose (often 300 mg) [9,10,11]. Clopidogrel’s half-life ranges from 7 to 8 h [12]. Clopidogrel is excreted almost equally by the kidney and the gastrointestinal system [13] (Figure 1). Based on in vitro data, approximately 85% of the orally administered drug is hydrolyzed by endogenous esterases into an inactive metabolite; the remaining drug is bioactivated in a two-step oxidative process by the cytochrome P450 (CYP450) enzyme in the liver to yield an intermediate metabolite (2-oxo-clopidogrel), which is then transformed to four isomeric thiol metabolites (H1–H4); clopi-H4 is the only active clopidogrel metabolite in vivo [14,15,16]. 

The active clopi-H4 metabolite irreversibly binds the purinergic adenosine diphosphate (ADP) platelet receptor P2Y12 [15], preventing platelet stimulation by ADP [11], subsequent platelet aggregation [7], and consequent thrombogenesis [8]. Under normal circumstances, P2Y12 and other platelet receptors bind to one arm of the fibrinogen molecule and link the fibrinogen molecule to receptors on other uninhibited platelets that bind the other arm of fibrinogen, thereby initiating aggregation. Translational studies consistently indicate the key roles of platelet aggregation, adhesion, and activation in the pathophysiology of thrombus formation and vessel occlusion that underlie coronary atherosclerosis [5,17,18,19,20,21,22]. Resultantly, rapid, reliable, and potent inhibition of platelets by drugs that bind the P2Y12 receptors on the surface of platelets is critical to decrease the frequency of ischemic events and thereby reduce cardiovascular morbidity and mortality [4,5].

Clopidogrel is most frequently paired with aspirin for dual antiplatelet therapy (DAPT) as an established standard of care for individuals with ischemic heart disease who have undergone PCI to reduce thrombotic risk [7,8]. Usually, postprocedural DAPT has a duration of up to 12 months, which depends on the type of stent placed during PCI and overall risk assessment [3], as well as shared decision-making with each individual patient. This follows a loading dose of clopidogrel given shortly before PCI. The preprocedural loading dose and the postprocedural DAPT therapy combination generally prevents stent thrombosis and limits ischemic events recurring after PCI [11]. Clopidogrel is in general well tolerated by individuals, with side effects mainly consisting of bleeding, gastrointestinal upset, rash, and other more rare adverse events such as hepatotoxicity or thrombotic thrombocytopenic purpura [11].

Unfortunately, some patients demonstrate limited response to the anti-platelet effect of clopidogrel. Platelet inhibition is attenuated in such patients, who exhibit “high on treatment platelet reactivity” (HTPR), which hinders optimal DAPT efficacy [11]. Platelets taken from patients that are tested ex vivo and found to be hyporesponsive to clopidogrel administration associate with a higher risk of ischemic coronary events, while platelets found to be hyperresponsive may associate with a higher risk of bleeding [16]. There is therefore interindividual variability in the observed response of individuals to clopidogrel therapy [23,24]. In fact, up to 30% of individuals presenting to health care facilities with ACS demonstrate poor response to clopidogrel therapy, in terms of HTPR and adverse cardiovascular endpoints [8,25].

Various clinical factors are thought to contribute to poor response to clopidogrel therapy as described in other sections of this text, while heritability appears to be responsible for up to 70% of interindividual variability [8,26]. As a result, genetic polymorphisms implicated in clopidogrel pharmacodynamics and pharmacokinetics, particularly absorption and metabolism, are considered key determinants of variable interindividual response to clopidogrel therapy [16]. 

## 2. Pharmacogenomics of Clopidogrel Response Variation

Approximately one fourth of individuals treated with clopidogrel exhibit a subtherapeutic response [27], and variable platelet reactivity in response to clopidogrel has been found to be highly heritable [11,26]. In fact, the expansive pharmacodynamics and pharmacokinetics of clopidogrel involving absorption in the intestine, bioactivation by CYP450 enzymes in the liver, and deactivation by esterases, provide various avenues for considering the basis of this heritability [11]. Such consideration could allow for discovery of differential metabolism of clopidogrel according to each individual’s genomic profile, with prediction of pharmacokinetic and pharmacodynamic responses and subsequent personalization of clopidogrel dosing in precision medicine [28]. This has led to a search for underlying genetic variants responsible for lack of efficacy, in order to apply pharmacogenomics to this clinical problem. Pharmacogenomics studies the impact of variations in the genome on response to therapeutics, with the goal of adjusting medication dosing to improve efficacy and minimize toxicity [28]. 

A range of 30–90% of variability noted in the pharmacokinetics and pharmacodynamics of therapeutics is considered attributable to pharmacogenomic variation [28]. Most pharmacogenomic studies over the past 20 years have involved enzymes that carry out oxidation, reduction, or hydrolysis of substrates (Phase I reactions) or enzymes that acetylate, glucuronidate, sulphate, or methylate their substrates (Phase II reactions) [28]. This includes human cytochrome P450 enzymes (CYP450) (mixed Phase I and Phase II reactions) [28], which have 57 active genes and 58 pseudogenes [11,29]. Genetic variants in *CYP2D6*, CYP2C19, CYP2C9 and *CYP3A4/5* are the most extensively studied [28,30]. The CY2D6 enzyme metabolizes several CYP450 substrates used in clinical medicine today, but is not a primary enzyme responsible for clopidogrel metabolism [28,30]. 

The CYP2C family (CYP2C8, CYP2C9, CYP2C18 and CYP2C19) metabolizes 20% of the substrates of CYP450 [31,32]. Genetic variants specifically in *CYP2C9* and *CYP2C19* associate with differential metabolism of various CYP450 substrates [28], such as clopidogrel [11,26,33,34]. Genetic variants in other CYP450 genes such as *CYP1A2* and *CYP2B6* can also influence the pharmacodynamics and pharmacokinetics of either the first or second step of clopidogrel metabolism [11,16,35,36,37]. Thus, for clopidogrel, investigations have largely focused on genomic variants associated with metabolism to its active metabolite by these major cytochrome P450 enzymes [11,27,28]. Clopidogrel metabolism can also be influenced by genetic variants in other proteins, such as the ATP-Binding Cassette Subfamily B Member 1 (*ABCB1*), Paraoxonase-1 (*PON1*), Carboxyl Esterase 1 (*CES1*), and P2Y12 receptors [11,16,35,36,37]. These genetic variants can interfere with regulation of gene transcription, protein translation, or substrate affinity [38,39].

The primary cytochrome P450 enzyme involved in the two-step bioactivation of clopidogrel has also arisen as the most highly associated with the variable response to clopidogrel: *CYP2C19* [1,11], which is therefore the focus of this review. The CYP2C19 enzyme is responsible for 45% of the first step and 20% of the second step in hepatic biotransformation of clopidogrel [40,41]. The enzymes CYP1A2 and CYP2B6 also assist in the first step, and CYP2B6, CYP2C9, CYP3A4 and CYP3A5 assist in the second step [11,40,42,43]. The CYP2C19 enzyme therefore plays an important role in both steps of this oxidative process. In a linear mixed-effects model using the AUC_0–24_ as a primary outcome based on active metabolite measurements in 162 normal subjects compiled from 6 separate studies, carriage of CYP2C19*2 or *3 was associated with the most significant reduction (32%, *p* < 0.001) in AUC_0–24_, compared to genetic variation in the other cytochrome P450 enzymes involved in clopidogrel metabolism, such as CYP2C9, CYP2B6, CYP3A5 and CYP1A2 [44]. Variants in *CYP2C19*, which encodes the enzyme involved in both the first and second steps have been linked to impaired bio-activation of clopidogrel, decreased platelet inhibition, and higher risk of adverse cardiovascular events following PCI [27]. 

### 2.1. CYP2C19 Variation

Interindividual variability in response to clopidogrel administration consists of clinical factors, and is also in part explained by genetic variants in *CYP2C19* [25]. Polymorphisms in *CYP2C19* at least in part account for variable production of the active clopidogrel metabolite (clop-H4). As a result, CYP2C19 has arisen as a key focus of investigation in clopidogrel pharmacogenetics and pharmacogenomics [23,45].

The enzyme cytochrome P450 2C19 (CYP2C19) is a 55.93 kDa (490 aa) protein encoded by the *CYP2C19* gene (90.21 kb) [28]. The gene is composed of 9 exons, which are mapped to the chromosomal location 10q24.1q24.3 [28]. The protein is expressed in liver cells, via 1901 bp, 2395 bp, and 1417 bp RNA transcripts [28]. CYP2C19 metabolizes a variety of drugs, including clopidogrel [46]. Heritable variation in CYP2C19 function was first noted 45 years ago [47]. Since then, more than 50 single nucleotide polymorphisms (SNPs) partially underlying variation in enzyme activity have been elucidated [48,49,50,51,52,53,54,55,56,57,58,59,60,61,62,63,64,65,66,67] (Table 1). The normal or wild type allele is usually denoted *1 (e.g., homozygous wild type *1/*1), with pathophysiological polymorphisms following similar nomenclature [11]. Enzyme activity is often assessed by its classical reaction, which produces (S)-mephenytoin 4′-hydroxylation [51].

#### 2.1.1. Loss-of-Function *CYP2C19* Variants 

The most extensively studied variants are CYP2C19*2 (primarily rs4244285) and CYP2C19*3 (rs4986893), which both involve replacement of guanine (G) with adenine (A) [11]. The CYP2C19*2 variant is present at nucleotide 681 (i.e., c.681G > A) found in exon 5 [11], and synonymously affects the amino acid proline at position 227; CYP2C19*3 is present at nucleotide 636 (i.e., c.636G > A) found in exon 3, and substitution of the tryptophan codon TGG with TGA [15,51,56]. Both variants result in a stop codon, indirectly via a cryptic aberrant splice site in the case of CYP2C19*2 and directly coded by TGA in the case of CYP2C19*3, and ultimately produce a truncated, nonfunctional, catalytically inactive CYP2C19 protein fragment [51,56]. Individuals who are homozygous for either CYP2C19*2 (i.e., *2/*2) or CYP2C19*3 (i.e., *3/*3) poorly metabolize CYP2C19 substrates [47], such as clopidogrel, contributing to inter-individual variability in therapeutic response [32]. Other alleles have also been associated with *CYP2C19* loss-of-function (LOF) (CYP2C19*4, *5, *6, *7 and *8); however, these variants are rare (<1% allelic frequency) and less studied [68,69]. 

#### 2.1.2. Gain-of-Function *CYP2C19* Variant

A gain-of-function variant has also been identified [25,70]: the variant CYP2C19*17 (rs12248560) involves replacement of cystine (C) with thymine (T) [64]. The CYP2C19*17 variant (i.e., g.−806C>T or g.−3402C>T) is present in a promotor region of the gene at the 5′-flanking region of exon 5 [25,71]. The genetic variant in the promoter region is thought to modify the interaction of transcription factors with the promotor region in *CYP2C19*, thereby altering the extent of transcription of the gene [71]. Thus, besides variation in the coding regions of the *CYP2C19* gene, variations in the promoter region of the gene also appears to influence variability [47]. The CYP2C19*17 allelic variant associates with higher levels of catalytic activity of the corresponding CYP2C19 enzyme, presumably due to increased transcription of the *CYP2C19* gene [11,25,64]. The activity of CYP2C19 depends on its level of expression in the liver, which is in part determined by regulation of gene transcription [46]. Thus, bioactivation of clopidogrel and other prodrugs to their therapeutic metabolites is increased in individuals carrying the CYP2C19*17 allele [15,72]. Consequently, individuals carrying the CYP2C19*17 allele have a higher risk of bleeding when treated with clopidogrel in the PLATO (Platelet Inhibition and Patient Outcomes) trial [25,73,74]. However, other studies have not demonstrated increased platelet inhibition or altered clinical outcomes with the CYP2C19*17 allele [26,75,76,77]. Interestingly, CYP2C19 enzyme activity levels in individuals homozygous for CYP2C19*17 overlaps the heterogeneous enzymatic activity noted in some wild type individuals [78]. This high enzyme activity in some individuals with wild type alleles could be due to other underlying causes of increased enzyme activity, such as response regulators described elsewhere in this article, or yet to be discovered gain of function alleles [47]. Therefore additional studies assessing the 5′-up-stream promotor region of the *CYP2C19* gene could also help clarify the observed heterogeneity of enzyme activity in some wild type individuals [47]. Nevertheless, in a study of livers from individuals heterozygous and homozygous for CYP2C19*17, transcribed mRNA levels were 1.8-fold and 2.9-fold higher, respectively, than livers with the homozygous wild type allele (*1/*1) [46]. Such studies may be consistent with the *17 allelic variant serving as a regulatory polymorphism that can enhance the expression of *CYP2C19* expression [46]. However, taken together, it is unclear how patients with the CYP2C19 *17 allele would respond to clopidogrel, and the combined effect of *2 and *17 on *CYP2C19* function and response to clopidogrel is uncertain.

#### 2.1.3. Linkage Disequilibrium

Of course, it is interesting to consider that in future studies the *17 gain-of-function variant could potentially compensate for the *2 or *3 loss-of-function variant [46]. Indeed, the CYP2C19*17 and CYP2C19*2 variants have been found to be in linkage disequilibrium, which could explain inconsistent accounts of the effect of the CYP2C19*17 variant on platelet aggregation and measured levels of the active clopidogrel metabolite (clopi-H4) [15,79,80]. Linkage disequilibrium has also been noted between the CYP2C19*2 and CYP2C19*35 variants. The CYP2C19*35 (rs12769205) involves alteration of adenine (A) at an intron 2 branch point, which results in an alternative CYP2C19 mRNA transcript in human liver that includes the entire intron 2 (exon 2B) [81]. The reading frame on the mRNA transcript is therefore altered, with the introduction of 87 additional amino acids and a premature termination or stop codon [81]. The CYP2C19*35 variant in isolation can decrease *CYP2C19* canonical mRNA transcript levels and subsequent translation of the CYP2C19 protein [81].

#### 2.1.4. Multiple Variants 

Most genes with variants that associate with poor clopidogrel response exert a weak on platelets inhibition [11]. However, several authors have suggested additive or synergistic effects on clopidogrel response in the context of coexistence of *CYP2C19* variants with polymorphisms in other genes [11,82,83,84]. For example, synergistic effects may be noted with coexistence of *CYP2C19* polymorphisms and *ABCB1* genetic variants in patients undergoing PCI [82]. Other studies suggest that coexistence of *CYP2C19* and *P2Y12* variants leads to a greater impact on clopidogrel response and clinical outcomes than either polymorphism alone [11,83,84]. As such, models to predict clopidogrel response should account for the effect of multiple variants in different proteins. Similar multimarker models have been developed in other areas of medicine with effective prediction of outcomes [85].

## 3. Pharmacogenomics Era

CYP2C19 genetic variants impact plasma levels of the active clopidogrel metabolite platelet response to clopidogrel [26,44].

### 3.1. Pharmacokinetic Phenotypes

Individuals with various combinations of the most common *CY2C19* variants (*1, *2, *3, *17) have been classified into at least four categories (Table 2) [11,47], to correspond to an observed gene-dose effect [25,86]. For example, individuals with allelic variants *2/*2, *3/*3, or *2/*3 can be considered “poor metabolizers (PM)”; individuals with variants *1/*2 or *1/*3, or likely also *2/*17 or *3/17 can be considered “intermediate metabolizers (IM)”; individuals with *1/*1 are considered wild type; and those with *17/*17 or *1/*17 may be considered “ultra-rapid metabolizers (UM)” [11,15,46,47]. These phenotypes were determined by association with interindividual variability in response to clopidogrel. To illustrate, individuals denoted as poor metabolizers (e.g., individuals with alleles homozygous for CYP2C19*2) or intermediate metabolizers (e.g., individuals with alleles heterozygous for CYP2C19*2, i.e., *1/*2) were noted to have an increased risk of ischemic events, in particular stent thrombosis [7,25,86,87] and intraprocedural thrombotic events during PCI [88]. Put simply, heterozygosity for loss-of-function alleles confers intermediate metabolism and homozygosity for loss-of-function alleles confers poor metabolism [25], while wild type alleles (*1/*1) confer the desired normal or “extensive” metabolism and homozygosity for gain-of-function alleles confers ultra-rapid metabolism. In European populations most often studied, the majority (~70%) of individuals are found to be non-carriers of CYP2C19 variants (besides the wild type *1/*1), while approximately 30% of individuals are intermediate metabolizers, and a small proportion ~2% are poor metabolizers carrying homozygous loss-of-function variants [28,86,89,90].

### 3.2. Impact on Platelet Function 

**Figure 2:**
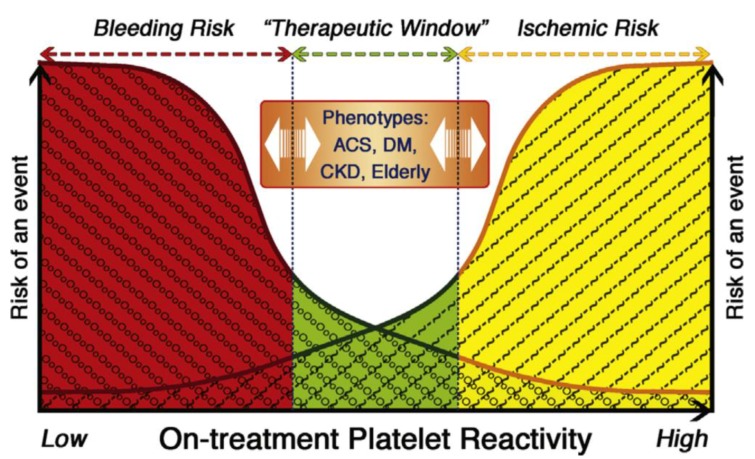
Variable platelet response to clopidogrel can associate with risk factors and outcomes. On-treatment platelet reactivity in response to clopidogrel can associate with bleeding risk if low or ischemic risk if high, with a therapeutic window. Various risk factors for ischemic heart disease can impact on-treatment platelet reactivity in individuals treated with clopidogrel. ACS: acute coronary syndrome; CKD: chronic kidney disease; DM: diabetes mellitus. Used with permission of Elsevier; copyright 2013 [107].

*CYP2C19* genetic variation can result in variable platelet inhibition (Figure 2). For example, reduced platelet inhibition by clopidogrel resulting from *CYP2C19* loss-of-function genetic variants was observed in several studies on platelet aggregation [11,25,26,32,44,91], with a gene-dose effect [87]. In general, platelet inhibition can be investigated in platelet function tests (PFTs) that can include bleeding time, platelet aggregation via light transmission, lumiaggregometry, whole blood impedance aggregometry, and/or use of flow cytometry to determine platelet activation [92]. Platelet aggregation is most often used to assess platelet inhibition by clopidogrel. A commonly used PFT assay is VerifyNow, which has been found effective for assessing platelet function in response to clopidogrel therapy [93]. VerifyNow measures optical signals from a whole-blood point-of-care cartridge containing fibrinogen-coated polystyrene beads, 20 μM ADP and 22 nM prostaglandin E_1_ to estimate the extent of platelet function inhibition in response to P2Y12 receptor blockade.

In some reports, HTPR in response to clopidogrel therapy affected up to 40% of treated individuals [9,94,95]. Of importance, the HTPR independently associated with larger intracoronary thrombus burden, worse post-PCI myocardial flow and perfusion, higher ischemic risk, and frequency of adverse cardiovascular outcomes such as stent thrombosis, myocardial infarction, stroke, and death in several studies on platelet aggregation, and therefore carries a poor prognosis in patients with ACS treated with clopidogrel after PCI [25,96,97,98,99,100]. HTPR can be partially overcome by assuring loading of clopidogrel prior to PCI, with administration of the higher dose (600 mg, compared to 300 mg) [101,102]. Of note, individuals with baseline high platelet reactivity prior to clopidogrel administration are more often identified as poor clopidogrel metabolizers over time [99]. Conversely, enhanced platelet inhibition by clopidogrel has been noted in association with the CYP2C19*17 gain-of-function allele [73,103]. 

Interestingly, attempts to tailor antiplatelet therapy based on PFTs were unsuccessful in regards to improving cardiovascular outcomes in the ARCTIC (Double Randomization of a Monitoring Adjusted Antiplatelet Treatment versus a Common Antiplatelet Treatment for DES Implantation, and Interruption versus Continuation of Double Antiplatelet Therapy) and ANTARCTIC (Platelet Function Monitoring To Adjust Antiplatelet Therapy In Elderly Patients Stented For An Acute Coronary Syndrome) clinical trials [104,105]. This illustrates the imperfect surrogacy of PFTs as a substitute for cardiovascular outcomes in studies assessing the impact of genetic variants on antiplatelet response to clopidogrel therapy. Several confounding factors interfere with these associations [11]. For instance, platelets reactivity dynamically changes with time and patient characteristics; frequent measurements may be necessary to determine and maintain personalized therapy [106].

### 3.3. Impact on Clopidogrel Metabolite Plasma Concentration

*CYP2C19* loss-of-function genetic variants in individuals who are poor metabolizers associate with lower plasma concentration of clopidogrel’s active metabolite clopi-H4 [34,44,91,103], which in turn can correlate with the extent of platelet inhibition [108,109,110]. The area under the plasma concentration curve over 0–24 h (AUC_0–24_) and maximum plasma concentration C_max_ for the active metabolite of clopidogrel for individuals with wild type *CYP2C19* (*n* = 56) were 76 ± 17.9 ng·h/mL and 58.4 ± 9.2 ng/mL, respectively, compared to CYP2C19*2 carriers, for whom the mean AUC_0–24_ and C_max_ were 41.5 ± 5.7 ng·h/mL and 35.3 ± 4.3 ng/mL, i.e., AUC_0–24_ was 54% and C_max_ was 60% that of individuals with wild type *CYP2C19* [34]. In this study, the AUC_0–24_ and C_max_ for one individual homozygous for CYP2C19*2, were 65% and 42% that of wild type individuals, respectively. A separate study included individuals with the CYP2C19*3 allele, as well as those with the CYP2C19*2 allele [103]. In that study, AUC_0–24_ and C_max_ for heterozygotes (*n* = 20) were 71% and 67% that of wild type individuals, respectively, and for homozygotes (*n* = 9) were 57% and 61% that of WT subjects for the active metabolite of clopidogrel, respectively [103]. The mean active clopidogrel metabolite concentration observed in patients with *CYP2C19* LOF alleles was lower by 0.14 μM·h, compared to extensive metabolizers, and approximately half the mean value seen in the overall study population (AUC_0–24_ = 0.35 μM·h) [87]. A randomized pharmacogenetics study suggested that a clopidogrel loading dose of 600 mg coupled with a maintenance dose of 150 mg led to partial restoration of measured levels of the active clopidogrel metabolite clopi-H4 to levels observed with the lower loading dose of clopidogrel 300 mg paired with the typical 75 mg maintenance dose in patients with wild type *CYP2C19* [16]. It is important to note that clopidogrel’s active metabolite clopi-H4 is relatively unstable; a stabilizing agent is added within 30 s of obtaining a blood sample [109], in order to avoid an incorrect assessment of clopidogrel metabolism. This poses one of many limitations to using the measured plasma concentration of clopidogrel’s active metabolite to evaluate the impact of genetic variants in *CYP2C19* [11,91,111].

### 3.4. Impact on Outcomes/Events

*CYP2C19* loss-of-function variants also associate with ischemic cardiovascular outcomes such as stent thrombosis and cardiovascular mortality, particularly in patients with acute coronary syndrome who undergo PCI, due to decreased enzyme expression and/or activity and consequent impairment of clopidogrel bioactivation [1,7,11,15,22,23,26,32,44,75,86,112], with an observed gene-dose effect [23]. For example, a genome-wide association study followed by CYP2C*2 genotyping in patients undergoing PCI suggested a link between the CYP2C*2 genotype and both diminished platelet response to clopidogrel (accounting for 12% of interindividual variation) and cardiovascular ischemic events or death during 1 year of follow-up (hazard ratio 2.42, *p* = 0.02) [26]. In another study, individuals carrying the CYP2C19*2 variant allele exhibited a 3-fold increased risk for adverse ischemic cardiovascular events on clopidogrel therapy [32,100]. Notably, this association is more prominent during the first year after PCI, and thereafter decreases significantly [113]. 

It should be noted that some studies suggest no relationship between *CYP2C19* loss-of-function genotypes and frequency of occurrence of ischemic cardiovascular events in individuals who are treated with clopidogrel [114]. This appears to depend on the patient population studied. For example, in the pharmacogenetic substudy of the CHARISMA (Clopidogrel for High Atherothrombotic Risk and Ischemic Stabilization, Management, and Avoidance) clinical trial, individuals with loss-of-function *CYP2C19* variants demonstrated no reduction in antiplatelet our outcome response to clopidogrel in this population of stable cardiovascular disease patients with no recent ACS or PCI [23]. Similar observations were made in another study that involved patients with atrial fibrillation, in which no correlation was seen between *CYP2C19* loss-of-function or gain-of-function variant status and cardiovascular outcomes in patients on clopidogrel [115]. In a collaborative meta-analysis that primarily focused on patients who underwent PCI, with data extracted from 9 previous studies involving 9685 study participants receiving clopidogrel (91.3% with PCI, 54.5% with ACS), carriers of 1 (Hazard Ratio (HR), 1.55; 95% Confidence Interval (CI), 1.11–2.17) or 2 (HR, 1.76; 95% CI, 1.24–2.50, *p* = 0.002) CYP2C19 LOF alleles had a significantly increased risk of composite endpoint (cardiovascular death, myocardial infarction, or stroke) [86]. Furthermore, a significantly increased risk in stent thrombosis was observed with carriers of one (HR, 2.67; 95% CI, 1.69–4.22, *p* < 0.0001) or two (HR, 3.97; 95% CI, 1.75–9.02, *p* = 0.001) *CYP2C19* LOF alleles [86]. In a meta-analysis evaluating 32 studies of 42,016 patients, a treatment-only analysis revealed that carriers of one or two *CYP2C19* LOF alleles were at a higher risk for cardiovascular events (relative risk 1.18, absolute risk increase of 8–12 events per 1000 individuals) [87]. When this analysis was restricted to studies with 200 or more events, the relative risk of increased events was not significant, and when confined to genetic studies nested within randomized trials, the *CYP2C19* genotype was not significantly associated with cardiovascular events. A limitation of this meta-analysis was the lack of specific analysis for patients undergoing stenting compared with other medical treatments, including a large number of patients who were being treated for reasons other than stenting (e.g., atrial fibrillation, STEMI). Therefore, there appears to be equipoise in the community regarding whether CYP2C19 *2 and *3 allele carriers are at increased risk for death or cardiovascular events when treated with clopidogrel therapy, especially after PCI. The heterogeneity of observations will continue to necessitate further investigation [32]. For instance, a large prospective *CYP2C19* genotype-based clinical trial, TAILOR-PCI, is currently ongoing, to address whether individualizing anti-platelet drug therapy based on genotype will attenuate outcomes [116,117].

## 4. Regulators of Response

Careful examination of a myriad of studies reveals inconsistency of the association of *CY2C19* loss-of-function or gain-of-function allelic variants with reduced antiplatelet activity, plasma clopidogrel active metabolite concentration, and ischemic cardiovascular outcomes, or the converse, respectively [15,23,73,76,103,118]. Some studies suggest that *CYP2C19* genetic polymorphisms are responsible for a sizeable genetic defect of up to 12% of the interindividual variability of platelet aggregation noted in individuals who have undergone PCI, and that >80% of the variability can be explained by other factors collectively [119]. Among reasons for such variability are the inability of various genotyping assays to detect all variants that possibly could affect CYP2C19 activity (although only a handful of variants potently impact enzyme activity), differential drug absorption among individuals, and drug–drug interactions [15]. The observation of genotype-phenotype discordance in some individuals can also imply differential regulation of the *CYP2C19* gene leading to an acquired deficiency in enzyme activity [47]. Indeed, antiplatelet response to clopidogrel administration may depend on genetic, cellular, environmental, and clinical factors [100].

**Figure 3:**
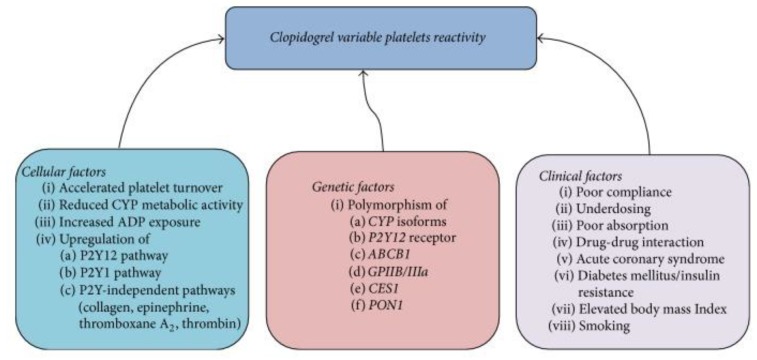
Conventional and Genetic Regulators of Response. Conventional clinical (and cellular) factors and also genetic other factors regulate response to clopidogrel. *ABCB1*: ATP-Binding Cassette Subfamily B Member 1; ADP: adenosine diphosphate; *CES*: Carboxylesterase 1; *CYP:* cytochrome P450, particularly CYP2C19; *GPIIB/IIIa*: Glycoprotein IIB/IIIa; *PON1*: Serum paraoxonase/arylesterase 1; P2Y: receptor on the surface of platelets. Used with permission of Creative Commons; copyright 2017 [11]; originally adapted from [128].

Platelet function and cardiovascular outcomes are affected not only by medical therapy and genetic variation, but also by non-genetic factors, such as concomitant diseases, patient compliance, obesity, age, race, sex, ethnicities, diet, smoking, platelet count, and hematocrit [11,23,32,92,120,121,122,123] (Figure 3). It should be noted that platelet reactivity levels and plasma concentration of active metabolites may vary with time and in different clinical settings, such as diabetes mellitus, chronic kidney disease, type of coronary lesions, stent-related factors, presentation with stable ischemic heart disease versus ACS, and inflammatory state (e.g., CRP or interleukin levels post-PCI [107,124,125,126]). Some studies suggest that high-sensitivity CRP and interleukin levels can predict response to clopidogrel [127], while other studies suggest that clopidogrel and other antiplatelet agents might be anti-inflammatory. Further, concomitant polymorphisms, differences in P2Y12 receptor density, concentration of fibrinogen and platelets, and accelerated turnover of platelets can also play a role [45,108]. Taken together, these observations elucidate the complex and nonlinear nature of the relationships among allelic variation, platelet reactivity, plasma concentration of active metabolite, and cardiovascular outcomes in the pharmacogenomics of CYP2C19 [16].

### 4.1. Variation by Ethnicity

Homozygosity for LOF *CYP2C19* variants is noted in approximately 2% of whites, 4% of blacks, and 14% of Chinese individuals [129] (Table 3). Heterozygosity for CYP2C19*2 is noted in up to 30% of Caucasians, up to 40% in African Americans, and up to 50% in East Asians [23,44,75,130]. The CYP2C19*3 allele is less common, and is found in less than 1% of self-reported Caucasians and African Americans and in 7–9% of Asians [11,15]. The CYP2C19*2 and *3 alleles are the most common and account for ≥99% LOF in a multi-ethnic population [68,69]. Of note, a high prevalence of LOF variants has been discovered in Vanuatu and Papua New Guinea, where allele frequencies are approximately 70% (*2) and 13% (*3) of *CYP2C19* alleles in Vanuatu, and approximately 40% and 30%, respectively, in Papua New Guinea, several orders of magnitude higher than in other ethnic populations [47,131,132]. The CYP2C19*17 allele also exhibits variation by ethnicity, with a low incidence of <5% in Japanese and Chinese populations, and higher a frequency of up to 30% in European and African populations [133].

Another study showed that African-Americans had a higher prevalence of CYP2C19*2 allele carrier status and higher on-treatment platelet reactivity than Europeans [134]. This study also suggested that African-American ethnicity and CYP2C19*2 allele carrier status were both independent predictors of HTPR. This implies that beyond CYP2C19*2 allele carrier status, additional factors may contribute to higher HTPR in African-Americans [46]. These factors may be genetic variants unique to African-Americans, conventional response regulators or response regulators based on a more comprehensive precision medicine approach described in subsequent sections. In addition, the CYP2C19*35 loss-of-function allelic variant is found in linkage disequilibrium with rs4244285 on CYP2C19*2 in whites, but is expressed in isolation in African-Americans [135]. On the other hand, a liver sample of African descent exhibited an approximately 12-fold increase in metabolic activity that was not accounted for by the presence of a CYP2C19*17 allelic variant, suggesting the existence of possible additional undiscovered variants or regulatory mechanisms [46].

One study identified lower frequency of the CYP2C19*2 variant in North Indians of Asian descent compared with the global population [136], while another study suggested comparable frequencies (40–47%) in Asian Indian patients with ischemic heart disease [137]. A meta-analysis showed that carrier status for LOF genetic variants in *CYP2C19* associated with increased risk of adverse clinical events [138]. The study also suggested higher risk of adverse clinical events in Asians compared to Western populations [138]. Paradoxically, although Japanese and other East Asian individuals have a higher incidence of *CYP2C19* LOF allelic variants than Europeans, Asians in general are reported to have a greater frequency of bleeding than Europeans [88,139]. The increased bleeding tendency observed in Asians, however, does not translate to less thrombotic events in Asians with *CYP2C19* LOF alleles receiving clopidogrel. For example, major cardiovascular and cerebrovascular events occurred at a significantly higher rate in Koreans and East Asians when treated with clopidogrel who had *CYP2C19* poor metabolizer status compared to wild type individuals [140,141].

### 4.2. Drug-Drug Interactions

Bioactivation of clopidogrel by CYP2C19 can be affected by drug-drug interactions [11]. The enzyme CYP2C19 also metabolizes several other drugs that are clinically useful, such as proton pump inhibitors (PPIs) (e.g., omeprazole, lansoprazole, and pantoprazole), selective serotonin reuptake inhibitors (e.g., citalopram and sertraline), tricyclic antidepressants (e.g., imipramine and amitriptyline), phenytoin, and clopidogrel [11,71]. In fact, there are almost 500 drugs that act as substrates (281), inhibitors (263), or inducers (23) of the CYP2C19 enzyme, or some combination of these.

Some studies suggest that PPIs inhibit the CYP2C19 enzyme, and that this may interfere with clopidogrel bioactivation to yield its active metabolite clopi-H4 [23,143,144]. Similar to carrying a LOF *CYP2C19* allele, co-administration of clopidogrel with certain PPIs leads to HTPR, and an approximately 40% increased risk in associated adverse cardiovascular outcomes and mortality, particularly in high-risk individuals [23,143]. Some PPIs exhibit a greater effect than others, such that omeprazole (the most potent CYP2C19 inhibitor) and lansoprazole, for example, associated with decreased platelet inhibition by clopidogrel, while esomeprazole and pantoprazole did not [11,23,143,145,146].

### 4.3. Conventional Regulators of Response 

#### 4.3.1. Adherence

As with any pharmacologic agent, patient adherence to the prescribed regimen is key to achieve optimal therapeutic effect and associated clinical outcomes. Studies indicate that nonadherence to prescribed clopidogrel therapy is a significant contributor to HTPR, but that nonadherence minimally accounts for the widely observed interindividual variability [91,147].

#### 4.3.2. Ischemic Heart Disease Risk Factors

It has been demonstrated that ischemic heart disease risk factors associate with low antiplatelet response to clopidogrel therapy [112] (Figure 2).

##### Sociodemographic Characteristics

Age (>65 years) and body mass index (BMI) are reported to account for approximately 20% of HTPR [26,148], and may be independent risk factors for reduced antiplatelet response to clopidogrel [112]. Individuals with effective response to clopidogrel have a BMI that is significantly less than the BMI for individuals with reduced antiplatelet response to clopidogrel therapy [149]. Some authors suggest weight-adjusting clopidogrel dosing [112].

##### Lifestyle Habits

Lifestyle habits such as diet, caffeine, and smoking can all increase platelet inhibition in response to clopidogrel, while grapefruit can interfere with platelet inhibition [11,150,151,152]. These effects are thought to be mediated by interaction with the CYP450 enzymes. 

##### Comorbid Conditions

Type 2 Diabetes Mellitus (DM2) appears to be an independent modulator of platelet response to clopidogrel [112]. HTPR is noted in patients with DM2 and associates with poor therapeutic outcomes [153,154], which may be overcome by administering a higher maintenance dose of clopidogrel such as 150 mg [11,155]. Some authors suggest BMI and DM2 status as more reliably predictive of HTPR and poor prognosis following PCI than other risk factors or genetic variant status alone [112]. In fact, it has been suggested that the poor metabolizer phenotype, BMI, and DM2 be combined as a more credible triple predictor of resistance to platelet inhibition by clopidogrel, and that these three can synergistically interfere with clopidogrel’s antiplatelet activity to incite major adverse cardiovascular events after PCI [112].

Individuals with chronic kidney disease (CKD) also demonstrate HTPR on clopidogrel [156] and increased adverse cardiovascular outcomes after PCI, in part related to elevated baseline platelet reactivity [157,158,159]. Furthermore, comorbid DM2 and CKD associate with HTPR on clopidogrel and poor outcomes [160]. Lipid levels also appear to account for approximately 20% of HTPR on clopidogrel [26]. Future studies on risk assessment should determine which and how many risk factors could be included in a multimarker predictor of clopidogrel resistance.

## 5. Multi-Omic Precision Medicine Approach to Response Regulation

**Figure 4:**
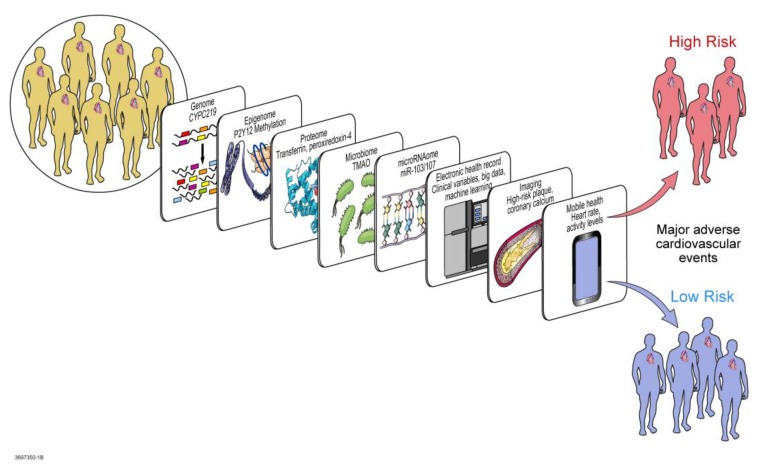
Systems Medicine tools for CYP450 regulation in Precision Cardiovascular Medicine. Studies in precision cardiovascular medicine have produced results from various omic technologies that help elucidate contributing factors regulating the pharmacogenomic impact of CYP450 variation on antiplatelet therapy. For example, systems biology tools have implicated *CYPC219* genomic variants in genome-wide association studies, methylation of P2Y12 in epigenomics, miR-103/107 on *CYP2C19* in microRNAomics, transferrin and peroxiredoxin-4 in proteomics, TMAO in metabolomics and microbiomics. Beyond omics, systems medicine and precision medicine incorporate ‘big data’ and clinical variables from the electronic health record with heart rate and activity levels from mobile health technology, along with findings from Imaging (such as high risk plaque, coronary calcium), to predict which individuals may be at low versus high risk for resistance to treatment with clopidogrel. CYP450: cytochrome P450; *CYP2C19*: cytochrome P450, family 2, subfamily C, polypeptide 19; *P2Y12*: the adenosine diphosphate receptor on the surface of platelets, to which clopidogrel binds; miR-103/107: microRNA-103 and microRNA-107; TMAO: trimethylene *N*-oxide.

Significant progress has been made in the diagnosis, prognosis, and therapeutics of ACS, yet initial and recurrent coronary events continue to plague some individuals especially after PCI. Pharmacogenomics has identified allelic variants in *CYP2C19* and other proteins involved in clopidogrel pharmacodynamics and pharmacokinetics that are associated with recurrent cardiovascular events. However, beyond conventional regulators of platelet response to clopidogrel therapy lie non-traditional regulators as described above that should be considered in precision cardiovascular medicine. Personalized care in precision medicine is not complete after pharmacogenomic testing and application alone. Precision medicine extends beyond genomics into multi-genomic marker models, transcriptomics, epigenomics, exposomics, miRNA regulomics, proteomics, metabolomics, microbiomics, and in silico computational and mathematical modeling, among other precision and systems medicine tools (Figure 4). Systems medicine applies systems biology to medicine. Systems biology studies whole organisms and networks by focusing not only on the details of each component, but also on how the system works together as a whole to respond to perturbations to produce emergent properties. As a result, a true precision approach to the pharmacogenomics of clopidogrel therapy considers various “omics” and other biotechnological tools resulting from, related to, and regulating pharmacogenomics. Precision cardiovascular medicine therefore integrates genomics with various other components of the human system and accounts for connectivity and interactions within the system as it responds to perturbation (in this case clopidogrel therapy) and develops emergent characteristics (in this case the observed clinical phenotype).

The new frontier in pharmacogenomics is likely to be the multidimensional interface between pharmacogenomics and various other components integrated in precision cardiovascular medicine. These components include not only the biotechnological tools, but also clinical factors unique to each individual, in the context of their environment and the resulting exposome. Undoubtedly the exposures influence each individual’s epigenomics, behaviors, and prognosis, all interacting with other omics. 

### 5.1. Transcriptomics

Transcriptomics looks at the complement of large-scale gene expression profiles at a particular timepoint while the organism is in its current state. In one study, analysis of specific mRNA transcripts in peripheral blood samples from a large community-based cohort identified platelet-derived inflammatory transcripts that associated with BMI; several transcripts were deemed heritable [161]. This supports associations previously noted between BMI and platelet reactivity [149]. Transcription can display tremendous variability in gene expression profiles in response to stimuli [27] (i.e., perturbations such as clopidogrel therapy). These profiles can be evaluated for discovery of grouped gene expression or pathways partially underlying variability in clopidogrel response. 

### 5.2. Epigenomics and Exposomics

Epigenomics involves modification of DNA via physical additions without change in DNA sequence, and includes methylation, acetylation, histone modification, and transcription factor binding, among other modifications. Several studies suggest that environmental characteristics or exposures such as pregnancy, old age, cancer, or congestive heart failure can modify CYP2C19 activity [162,163,164,165]. Such modifications can contribute to genotype-phenotype discordance [47]. This could occur via CpG methylation, for example, or by influencing the expression or activity of transcription factors, or post-translational regulation mechanisms [47]. Physical binding of transcription factors to promotor regions to initiate gene transcription can be affected by methylation of CpG motifs in the promoter region or other location in the gene to be transcribed [47,166]. For example, methylation status at three P2Y12 promoter sites (CpG11, CpG12, and CpG13) correlated with HTPR and an increased risk of ischemic events in patients treated with clopidogrel [167,168]. Epigenomic modification can silence an allele and result in preferential expression of the complementary allele. This phenomenon, known as allelic expression imbalance, may contribute to CYP2C19 expression levels [47]. Further, DNA methylation throughout the genome and acetylation of chromatin to modify histone binding can be heritable [27,169].

### 5.3. miRNA Regulomics

MicroRNAs (or miRNAs) are small non-coding RNAs that suppress protein translation by directly binding to mRNA transcripts for the affected protein [22]. These miRNAs can promiscuously bind various transcripts in related biological networks and pathways, thereby having profound pathophysiological effects that can fine-tune complex biological processes [27,170]. Thus, miRNAs can post-transcriptionally regulate CYP2C19 expression, as predicted by in silico modeling and simulation [171] and confirmed experimentally [172]. The miRNA combination miR-103/107 binds the 3′UTR of *CYP2C19* at 222–242 bp and 138–152 bp down-stream of a stop codon [172]. Thus, *CYP2C19* post-transcriptional regulation may contribute to interindividual variability in response to clopidogrel therapy [47].

### 5.4. Proteomics

Once mRNA transcripts are translated, the protein products can be measured on a large scale in a proteomics approach. In a study, the proteome of platelets was interrogated 24 h after administration of the clopidogrel loading dose [173]. Higher levels of the platelet adhesion molecule cluster differentiation-226 (CD226) and transferrin, and lower levels of peroxiredoxin-4, associated with HTPR on clopidogrel [173]. Since proteins also may undergo chemical modifications such as phosphorylation, nitrosylation, or acetylation, to regulate structure and function, the use of proteomics also aims to identify such chemical modifications associated with some perturbation of the studied system [27].

### 5.5. Metabolomics and Microbiomics

Metabolomics studies the small molecule compounds (often active metabolites) that result from biotransformation of prodrugs like clopidogrel. An organism’s metabolome varies with perturbation of the system by environmental factors, drugs, or other modalities [174]. The metabolomic analysis can yield a metabolic fingerprint or metabotype that discriminates an individual’s response to the perturbation, such as association of metabolic biomarkers of drug response with variable platelet reactivity on clopidogrel therapy [11,175]. Thus, metabolomics or pharmacometabolomics can partner with pharmacogenomics to potentially predict cardiovascular outcomes on clopidogrel therapy [11]. In the same way, microbiomics can also partner with pharmacogenomics. A poignant example involves trimethylamine *N*-oxide (TMAO), which is a small and colorless amine oxide produced by microbial metabolism [176]. The metabolite TMAO is derived from dietary phosphatidylcholine, and also from carnitine and betaine, by gut microbes [176,177]. Studies have now linked high levels of TMAO with platelet hyperactivity and incident thrombosis [178], and consequently increased risk for cardiovascular events [177]. Even after adjusting for conventional cardiovascular risk factors and risk biomarkers such as high-sensitivity C-reactive protein, TMAO levels predict all-cause mortality risk [179]. Notably, administration of aspirin appears to attenuate the effect of TMAO on platelets [179], likely due to observed effects of aspirin on composition of the gut microbiota [180]. Conversely, high levels of TMAO may antagonize the antiplatelet activity of aspirin, as well as clopidogrel [179]. This raises an important issue for both clopidogrel and aspirin in DAPT, which is independent of *CYP2C19* variation and can potentially synergize the effect of the LOF *2 and *3 alleles or counteract any effect of gain-of-function alleles such as possibly *17.

### 5.6. Mathematical, Computational, and Molecular Modeling

Mathematical, computational, and molecular modeling can be used to simulate clopidogrel pharmacodynamics and/or pharmacokinetics in silico. In a dynamic pharmacokinetic model, developed for clopidogrel and its active metabolite clopi-H4, four CYP2C19 metabolic phenotypes are simulated: poor, intermediate, extensive, and ultra-rapid metabolizers [181]. Each simulated phenotype virtually receives a clopidogrel loading dose of 300 mg, then a maintenance dose of 75 mg. Several approaches were used to validate the model, which accurately predicted pharmacokinetics of clopidogrel and its intermediate and final active metabolite in the four phenotypic metabolism groups [181]. Another model simulated in silico epigenomics to evaluate and predict the function of binding sites in *CYP2C19* that could potentially regulate antiplatelet response to clopidogrel.

## 6. Toward Implementation in Clinical Practice

DAPT therapy remains pivotal for patients undergoing PCI [47]. However, interindividual variability in platelet reactivity and clinical response to clopidogrel lead to therapeutic failure in some patients [47]. These observations have led to extensive searches on a variety of causes of treatment failure [22]. Some of the variability is determined by interacting inducer and inhibitor drugs [182]. Minimal effects on variability are associated with genetic variants in *PON1*, *CYP3A4/5*, *ABCB1*, *P2Y12*, and *CES1* [11,33,35,36,37,183].

Clinical and pharmacogenomic (primarily involving *CYP2C19*) factors causes have been identified, but these account for only approximately 20% of clopidogrel pharmacokinetics and up to 65% of clopidogrel pharmacodynamics [108]. Allelic variants alone in *CYP2C19* account for only 12% of clopidogrel variability in platelet reactivity [26,184]. Thus, the underlying pathophysiology of a great proportion of the etiology of clopidogrel HTPR has not yet been elucidated [47]. 

Pharmacogenomic testing is commercially available and often reimbursable for *CYP2C19* genotyping [11,27]. Of note, some commercial target arrays measure only the common SNPs, e.g., *2, *3, and *17. The absence of these SNPs reflexes the report to wild type, i.e., *1. This could be misleading clinically, as other LOF genotypes (however rare) such as *4–*7 and *20–*21, are misclassified as normal. In the future, next generation sequencing, which would assess the entire *CYP2C19* gene sequence, may be useful to more precisely identify CYP2C19 variants in clinical settings. Nevertheless, most physicians have not begun to order or act on such testing [11]. Consequently, additional studies would be needed to assess cost-effectiveness and best practice methods for implementation and delivery, likely requiring specialized physician education and utilizing systematic clinical decision support tools [11], for shared decision-making sessions with patients. Findings from some studies suggest that genotyping *CYP2C19* may be cost-effective for personalization of clopidogrel therapy [7]. 

**Figure 5:**
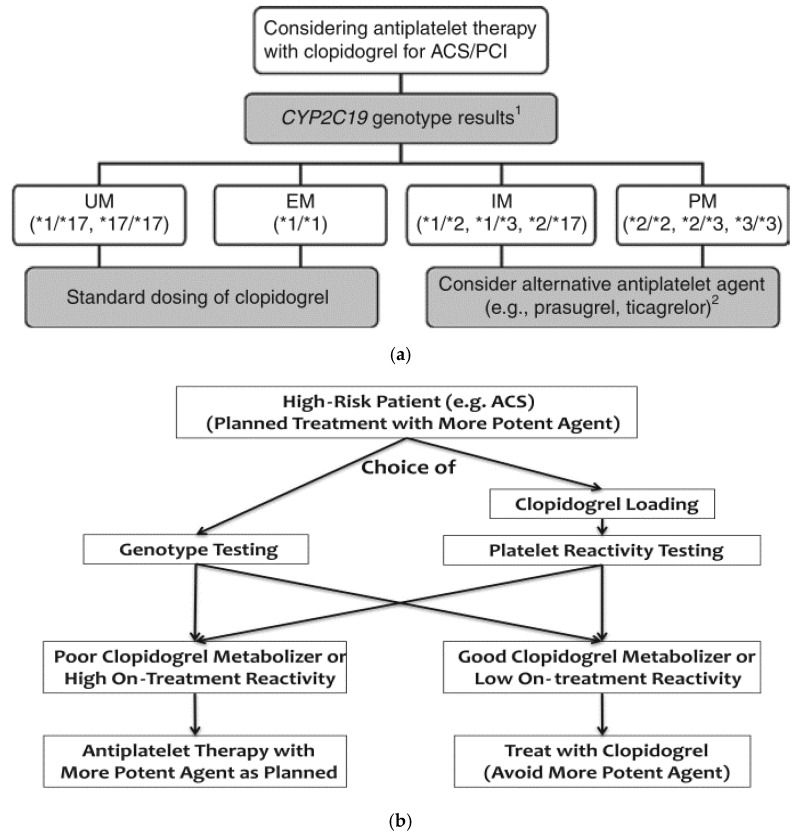
Proposed Clinical Algorithms for Tailoring Clopidogrel Therapy. Proposed clinical algorithms for tailoring clopidogrel therapy based on *CYP2C19* genotype (**a**,**b**) or on-treatment platelet reactivity (**b**). The algorithms do not account for non-genetic or other precision medicine regulators of clopidogrel resistance. ^1^ Only the most common *CYP2C19* genotypes are illustrated; ^2^ Prasugrel and ticagrelor should be considered when not contraindicated clinically. ACS: acute coronary syndrome; EM: extensive metabolizer; IM: intermediate metabolizer; PCI: percutaneous coronary intervention; PM: poor metabolizer; UM: ultra-rapid metabolizer. Used with permission of John Wiley and Sons; copyright 2013 by Scott et al. Clinical Pharmacogenetics Implementation Consortium guidelines for *CYP2C19* genotype and clopidogrel therapy: 2013 update. [68]; and used with permission of Elsevier; copyright 2017 [107].

It has been inferred that perhaps patients with *CYP2C19* LOF alleles should be warned about their probabilistic risk for adverse cardiovascular outcomes on clopidogrel following PCI [32]. If so, a higher dose of clopidogrel has been proposed as an option in attempts to overcome clopidogrel resistance [76]. However, in the GRAVITAS/GIFT trial, an increased clopidogrel maintenance dose of 150 mg daily (with a loading dose of 600 mg) as compared to 75 mg did not seem to overcome the risk of HTPR in CYP2C19*2 carriers [185] (add 2011 GRAVITAS reference also). In the ELEVATE-TIMI 56 trial demonstrated that a higher maintenance clopidogrel dose of 225 or 300 mg significantly reduced the number of CYP2C19*2 heterozygotes who had HTPR from 52% to 10% (*p* < 0.001), but homozygotes remained resistant at a dose as high as 300 mg [186]. The long-term safety of maintenance clopidogrel doses at 225 or 300 mg (or potentially of loading doses >600 mg) is unknown and cannot yet be safely recommended. Prasugrel and ticagrelor as alternatives to clopidogrel would be reserved for poor and intermediate metabolizers [7] (Figure 5). This would be in keeping with guidelines from the Clinical Pharmacogenetics Implementation Consortium (CPIC), which recommend *CYP2C19* genotyping in individuals with ACS undergoing PCI, and alternative antiplatelet therapy with prasugrel or ticagrelor for individuals with 1 or 2 *CYP2C19* LOF alleles (i.e., intermediate or poor metabolizers, respectively) [15,72]. The CPIC guidelines have been useful for standardization and consistency at various institutions involved in a network collaboration (The Translational Pharmacogenetics Program; TPP) funded by the National Institutes of Health (NIH) Pharmacogenomics Research Network [69]. The TPP is tasked with developing strategies for real-world dissemination and implementation of pharmacogenomics and assists with interpretation of alleles such as those salient to treatment with clopidogrel. 

Determining the *CYP2C19* genotype for each patient could therefore have utility [32]. Yet, there has been marginal translation of this potential utility into clinical practice [47]. This is in large part due to insufficient precision in prediction of response to clopidogrel and lack of prospective data demonstrating benefit in treating patients with *CYP2C19* LOF alleles with alternative anti-platelet therapy [47,187]. The probable value of routine pharmacogenetic testing in patient on clopidogrel therapy is still uncertain [25]. Thus, *CYP2C19* genotyping, or phenotypic testing with platelet reactivity or clopidogrel active metabolite concentration, does not yet carry enough evidence to guide therapeutic management [99].

The United States Food and Drug Administration issued a black box warning regarding the use of clopidogrel [41]. The statement suggested genotype testing to identify patients who may be *CYP2C19* poor metabolizers, obviating consideration of alternative therapies. However, routine testing is not yet recommended by the American College of Cardiology (ACC), American Heart Association (AHA), or Society for Cardiovascular Angiography and Interventions (SCAI) [2,3,15,41,188]. Adequately sized clinical trials would be needed to determine the effectiveness of routine pharmacogenetic testing for clopidogrel therapy [11]. To address this persistent need, two separate large prospective randomized clinical trials, TAILOR-PCI and the POPular Genetics study are currently ongoing [15,117,189]. Data from such large prospective randomized clinical trials could be incorporated into predictive mechanisms on the horizon. Proposed algorithms (e.g., Figure 5), may then shed light on pathways for patients with perplexing combinations of CYP2C19 polymorphism and platelet reactivity. This would apply for patients potentially presenting with genetic variants suggesting poor clopidogrel metabolism but with observation of low platelet reactivity, or patients with variants suggesting rapid clopidogrel metabolism but with observation of high platelet reactivity. The algorithm currently proposes a choice of either genotype testing or platelet reactivity testing, with only one type of test guiding therapy.

**Figure 6:**
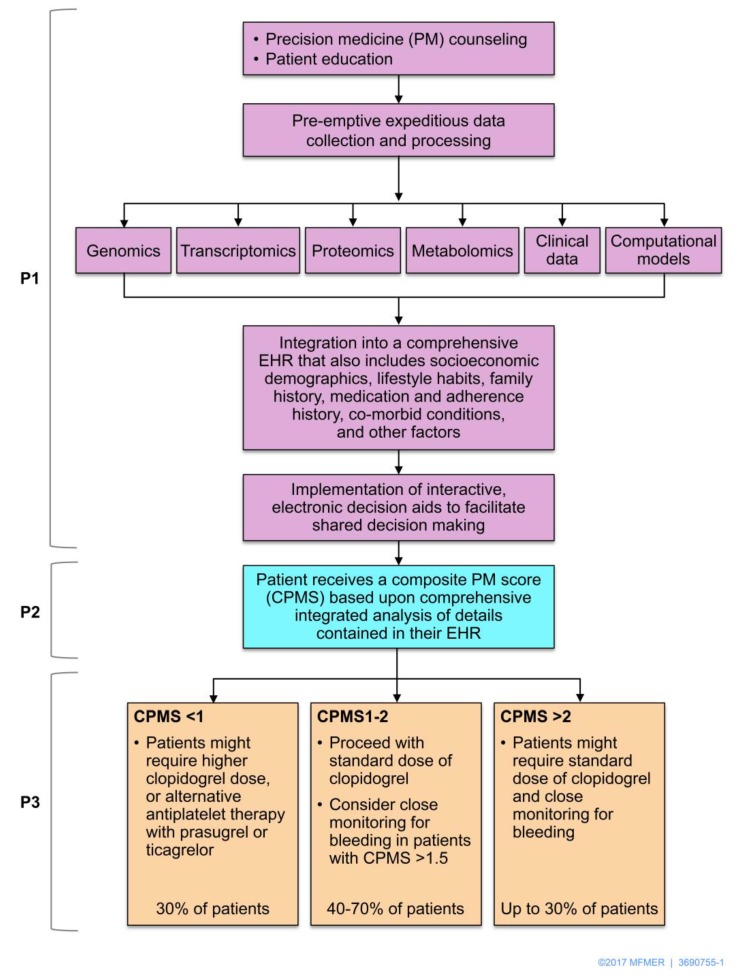
The P^*^3 precision medicine approach to tailoring antiplatelet therapy. In the P^*^3 pathway for incorporating precision medicine (PM) data in clinical practice, pre-emption (P1) is depicted in purple, prediction (P2) is depicted in blue, and prevention (P3) is depicted in brown. In P1, precision medicine data should be integrated with socioeconomic demographics, lifestyle habits, family history, medication and adherence history, co-morbid conditions, and other factors in the electronic health record (EHR). Clinical decision aids could support shared decision-making. A composite precision medicine score (CPMS) synthesizes clinical factors, platelet reactivity, and other test results with precision medicine data (such as genotype information) for risk prediction. In the example of clopidogrel, the integrative CPMS could be normalized to produce three risk categories (high, intermediate, and low) as shown. In P3, individualized prevention strategies would aim to maximize efficacy, as well as safety (e.g., with monitoring for any clinical evidence of bleeding). Thus, clopidogrel dosing or alternative antiplatelet therapy would not only depend on assessing *CYP2C19* genotype and/or platelet reactivity. Used with permission of the Nature Publishing Group; copyright 2015.

Indeed, determining precise methods to predict response to clopidogrel therapy is crucial to guide antiplatelet therapy in the future [11]. Developing such precision would depend on tailoring treatment based on each individual patient’s genetic and non-genetic clinical information [190]. Truly personalized care in precision cardiovascular medicine will necessitate integration of genomics, transcriptomics, epigenomics, exposomics (based on exposures), miRNA regulomics, proteomics, metabolomics, microbiomics, mathematical and computational modeling, to prevent, diagnose, prognosticate, and manage disease [191]. A systems biology approach in precision medicine [85] will likely be the next frontier of clopidogrel therapy personalization [11] (Figure 6).

## Figures and Tables

**Table 1:** Variants of *CYP2C19*. Used with permission of Creative Commons; copyright 2012 [47].

Allele	Characteristic SNP ^a^			Functional Change	References
	cDNA	Gene	Effect		
CYP2C19*1	None ^1^	None	None	Normal	[49]
CYP2C19*2	681G>A ^2^	19154G>A	Splicing defect	Non-functional	[50,51,52,53,54]
CYP2C19*3	636G>A ^3^	17948G>A	Premature stop codon (W212X)	Non-functional	[52,55]
CYP2C19*4	1A>G ^4^	1A>G	GTG initiation codon	Non-functional	[56,57]
CYP2C19*5	1297C>T ^5^	90033C>T	R433W	Non-functional	[58,59]
CYP2C19*6	395G>A	12748G>A	R132O	Non-functional	[51]
CYP2C19*7		19294T>A	Splicing defect	Non-functional	[60]
CYP2C19*8	358T>C	12711T>C	W120R	Decreased in vitro	[60]
CYP2C19*9	431G>A	12784G>A	R144H	Decreased in vitro	[61]
CYP2C19*10	680C>T	19153C>T	P227L	Decreased in vitro	[61]
CYP2C19*11	449G>A	12802G>A	R150H	Similar to wild type in vitro	[61]
CYP2C19*12	1473A>C	90209A>C	X491C; 26 extra amino acids	Unstable in vitro	[61]
CYP2C19*13	1228C>T	87290C>T	R410C	Similar to wild type in vitro	[61]
CYP2C19*14	50T>C	50T> C	L17P	Not determined	[61]
CYP2C19*15	55A>C	55A>C	I19L	Not determined	[61]
CYP2C19*16	1324C>T ^6^	90060C>T	R442C	Not determined	[62]
CYP2C19*17		3402C>T		Increased transcription in vitro, should not be termed Ultrarapid (UM)	[63]
		−806C>T			
CYP2C19*18	986G>A	80156G>A	R329H	Not determined	[51]
		87106T>C			
CYP2C19*19	151A>G	151A>G	S51G	Not determined	[51]
		87106T>C			
CYP2C19*20 ^7^	636G>A	17948G>A	Premature stop codon (W212X) and D360N	Non-functional	[51]
CYP2C19*21 ^8^	681G>A	19154G>A	Splicing defect and A161P	Non-functional	[51,54]
		−98T>C			
CYP2C19*22	557G>C	17869G>C	R186P and G91R	Not determined	[64]
CYP2C19*23	271G>C	12455G> C	R335O	Not determined	[65]
CYP2C19*24	1004G>A	80174G>A	F448L	Not determined	[65]
	1197A>G	87259A>G			
CYP2C19*25	1344C>G	90080C>G	D256N	Not determined	[65]
CYP2C19*26	766G>A	19239G>A	V374I	Decreased in vitro	[53]
CYP2C19*27		−1041G>A		Decreased in vitro	[66]
CYP2C19*28	1120G>A	−2020C>A		No significant decrease in vitro	[66]
		−1439T>C			
		80290G>A			

^a^ Only major single nucleotide polymorphism (SNP) or alteration(s) responsible for the phenotype of the corresponding allele is shown. Adapted from http//www.cypalleles.ki/se/; ^1^ The presence of additional SNP can further sub-classify individuals as *1B (99C>T; 991A>G) or *1C (991A>G). This results in an I331V change but does not alter activity; ^2^ The presence of additional SNP can further sub-classify individuals as *2A, *28, *2C, and *2D. Of these variants *2C and *20 harbor a SNP in the 5′ promoter region (-98T>C) that may have a functional effect; ^3^ The presence of additional SNP can further sub-classify individuals as *3A (1251A>C) and *3B (1078G>A; 1251A>C); ^4^ The presence of -3402C>7; -806C>T SNP in the promoter can further sub-classify individuals as *4B; ^5^ The presence of 99C>T; 991A>G, can further sub-classify individuals as *5B;^6^ Existence of the CYP2C19*2 polymorphism 681G>A on the same allele cannot be excluded; ^7^ Also known as CYP2C19*38; ^8^ Also known as CYP2C19*2C.

**Table 2:** Assignment of likely CYP2C19 phenotypes based on genotypes. Adapted from [68], used with permission of John Wiley and Sons; copyright 2013.

Phenotype	Example Genotypes	Enzyme Activity
Ultra-rapid metabolizer (UM)	*1/*17	Normal or increased
	*17/*17	
Extensive metabolizer (EM)	*1/*1 (wild type)	Normal
Intermediate metabolizer (IM)	*1/*2	Intermediate
	*1/*3	
	*2/*17	Likely intermediate
	*3/17	Likely intermediate
Poor metabolizer (PM)	*2/*2	Low or absent
	*3/*3	
	*2/*3	

**Table 3:** Variant and allele frequencies in the human *CYP2C19* family among various ethnicities. Adapted from [49], used with permission of Creative Commons; copyright 2017.

Allele	Defining Variants	Variant Type	Allele Frequencies in Indicated Populations, %					Functional Consequence
			EUR	AFR	EAS	SAS	AMR	
*1	None			59.2	44.5	60.5	51.9	77
*2	rs4244285	Splicing defect	18.3	18.1	31	34	10.1	Inactive
*3	rs4986893	Stop-gain (W212X)	<0.1	<0.1	6.7	0.4	<0.1	Inactive
*4	rs28399504	Start lost	0	<0.1	<0.1	<0.1	0.2	Inactive
*5	rs56337013	Missense (R433W)	0	0	0	<0.1	0	Inactive
*6	rs72552267	Missense (R132Q)	0	0	<0.1	0	<0.1	Inactive
*7	rs72558186	Splicing defect	0	0	0	<0.1	0	Inactive ^b^
*8	rs41291556	Missense (W120R)	<0.1	<0.1	0	<0.1	<0.1	Inactive
*9	rs17884712	Missense (R144H)	0	1.2	0	<0.1	<0.1	
*10	rs6413438	Missense (P227L)	0	0.4	<0.1	0	<0.1	Decreased ^a^
*12	rs55640102	Stop-lost (X491C)	0	<0.1	0	0	0	Decreased ^a^
*13	rs17879685	Missense (R410C)	0	1.6	0	<0.1	0.1	
*15	rs17882687	Missense (I19L)	0	2	0	<0.1	<0.1	
*16	rs192154563	Missense (R442C)	0	<0.1	0	<0.1	0	
*17	rs12248560	Regulatory	22.4	23.5	1.5	13.6	12	Increased
*22	rs140278421	Missense (R186P)	0	0.1	0	0	<0.1	
*23	rs118203756	Missense (G91R)	0	0	<0.1	0	0	
*24	rs118203757	Missense (R335Q)	0	<0.1	0	<0.1	<0.1	
*25	rs118203759	Missense (F448L)	0	0	0	0	0	
*27	rs7902257	Regulatory	0.1	8.3	0.1	0	0.3	Decreased ^a^

AFR, Africans; AMR, admixed Americans; CYP, cytochrome P450; EAS, East Asians; EUR, Europeans; SAS, South Asians. For references describing the functional characterization of the indicated alleles, see [142]. ^a^ Indicates alleles whose functionality assessment is based solely on in vitro data. ^b^ Indicates alleles whose functionality assessment is based solely on in vivo data.
